# Correction: Multimorbidity, polypharmacy, and COVID-19 infection within the UK Biobank cohort

**DOI:** 10.1371/journal.pone.0251613

**Published:** 2021-05-06

**Authors:** Ross McQueenie, Hamish M. E. Foster, Bhautesh D. Jani, Srinivasa Vittal Katikireddi, Naveed Sattar, Jill P Pell, Frederick K. Ho, Claire L. Niedzwiedz, Claire E. Hastie, Jana Anderson, Patrick B. Mark, Michael Sullivan, Catherine A. O’Donnell, Frances S. Mair, Barbara I. Nicholl

The Funding statement is incorrect. The correct Funding statement is as follows: HF was funded by a Medical Research Council Clinical Research Training Fellowship (grant reference number MR/T001585/1). The remaining authors received no specific funding for this work.
