# Supplementary material for: Multimorbidity, polypharmacy, and COVID-19 infection within the UK Biobank cohort
Source: PLoS One. 2020 Aug 20;15(8):e0238091. doi: 10.1371/journal.pone.0238091 (PMC7440632; doi:10.1371/journal.pone.0238091)
Supplement: S1 Table — (DOCX) [file pone.0238091.s001.docx]

## S1 Table – Cohort characteristics by COVID-19 testing.

|  | **Not tested for COVID-19  (n=423,701)** | **Tested for COVID-19  (n=4,498)** |
| --- | --- | --- |
| **Sex** | | |
| Female | 232,834 55.0 % | 2,301 51.2 % |
| Male | 190,867 45.0 % | 2,197 48.8 % |
| **Age at time of COVID-19 testing (years)** | | |
| 48-59 | 93,950 22.2 % | 1,083 24.1 % |
| 60-69 | 138,992 32.8 % | 1,132 25.2 % |
| 70-86 | 190,759 45.0 % | 2,283 50.8 % |
| **Ethnicity** | | |
| White | 396,471 94.1 % | 4,056 90.7 % |
| Asian or Asian British | 9,108 2.2 % | 138 3.1 % |
| Black or black British | 7,559 1.8 % | 167 3.7 % |
| Chinese | 1,393 0.3 % | 9 0.2 % |
| Mixed | 2,628 0.6 % | 33 0.7 % |
| Other ethnic group | 4,136 1.0 % | 68 1.5 % |
| **Townsend quintile** | | |
| 1 (least deprived) | 84,335 19.9 % | 684 15.2 % |
| 2 | 85,937 20.3 % | 780 17.4 % |
| 3 | 85,217 20.1 % | 793 17.7 % |
| 4 | 84,738 20 % | 954 21.2 % |
| 5 (most deprived) | 82,981 19.6 % | 1,279 28.5 % |
| **Smoking status** | | |
| Never | 233,534 55.4 % | 2,164 48.5 % |
| Current or Previous | 187,670 44.6 % | 2,298 51.5 % |
| **Frequency of alcohol intake** | | |
| Never or special occasions only | 81,997 19.4 % | 1,151 25.7 % |
| One to three times a month | 47,188 11.2 % | 530 11.8 % |
| One to four times a week | 206,681 48.9 % | 1,928 43.1 % |
| Daily or almost daily | 86,551 20.5 % | 869 19.4 % |
| **BMI (kg/m^2^)** | | |
| <18.5 | 2,114 0.5 % | 31 0.7 % |
| 18.5-25 | 134,711 32 % | 1,149 25.9 % |
| 25-30 | 180,942 43.0 % | 1,852 41.7 % |
| 30-35 | 74,585 17.7 % | 898 20.2 % |
| >35 | 28,870 6.9 % | 512 11.5 % |
| **Physical activity level** | | |
| none | 25,581 6.1 % | 463 10.6 % |
| low | 15,523 3.7 % | 215 4.9 % |
| medium | 333,409 79.8 % | 3,323 75.9 % |
| high | 43,127 10.3 % | 376 8.6 % |
| **Number of long-term conditions** | | |
| 0 | 147,988 35.1 % | 1,189 26.6 % |
| 1 | 139,061 32.9 % | 1,287 28.8 % |
| ≥2 | 135,094 32.0 % | 1,986 44.5 % |
| **Number of cardiometabolic long-term conditions** | | |
| 0 | 298,453 70.4 % | 2,683 59.6 % |
| 1 | 102,294 24.1 % | 1,285 28.6 % |
| ≥2 | 22,954 5.4 % | 530 11.8 % |
| **Number of respiratory long-term conditions** | | |
| 0 | 370,350 87.4 % | 3,796 84.4 % |
| 1 | 50,778 12.0 % | 634 14.1 % |
| ≥2 | 2,573 0.6 % | 68 1.5 % |
| **Number of medications** | | |
| 0 | 120,597 28.5 % | 987 22 % |
| 1 – 3 | 196,033 46.3 % | 1,789 39.9 % |
| 4 – 6 | 75,537 17.9 % | 1,026 22.9 % |
| 7 – 9 | 22,471 5.3 % | 438 9.8 % |
| ≥ 10 | 8,361 2.0 % | 249 5.5 % |
