# Supplementary material for: Multimorbidity, polypharmacy, and COVID-19 infection within the UK Biobank cohort
Source: PLoS One. 2020 Aug 20;15(8):e0238091. doi: 10.1371/journal.pone.0238091 (PMC7440632; doi:10.1371/journal.pone.0238091)
Supplement: S2 Table — (DOCX) [file pone.0238091.s002.docx]

## S2 Table – Relative risk of COVID-19 testing by multimorbidity (Poisson regression).

| **Measure of Multimorbidity (n)** | **Model 1**  **RR (95% CI)** | **P value** | **Model 2**  **RR (95% CI)** | **P value** |
| --- | --- | --- | --- | --- |
| **Total number of LTCs** | | | | |
| 0 (149,177) | 1 (ref) |  | 1 (ref) |  |
| 1 (140,348) | 1.14 (1.05-1.23) | *** | 1.10 (1.01-1.19) | 0.03 |
| ≥2 (137,080) | 1.73 (1.61-1.87) | *** | 1.54 (1.42-1.67) | *** |
| **Number of cardiometabolic LTCs** | | | | |
| 0 (301,136) | 1 (ref) |  | 1 (ref) |  |
| 1 (103,579) | 1.32 (1.23-1.41) | *** | 1.24 (1.15-1.33) | *** |
| ≥2 (23,484) | 2.20 (2.00-2.43) | *** | 1.88 (1.70-2.09) | *** |
| **Number of respiratory LTCs** | | | | |
| 0 (374,146) | 1 (ref) |  | 1 (ref) |  |
| 1 (51,412) | 1.21 (1.11-1.32) | *** | 1.14 (1.05-1.25) | *** |
| ≥2 (2,641) | 2.30 (1.81-2.93) | *** | 1.94 (1.51-2.49) | *** |
| **Number of medications** | | | | |
| 0 (121,584) | 1 (ref) |  | 1 (ref) |  |
| 1 – 3 (192,822) | 1.13 (1.05-1.22) | 0.05 | 1.10 (1.02-1.19) | 0.02 |
| 4 – 6 (76,563) | 1.61 (1.47-1.77) | *** | 1.47 (1.34-1.62) | *** |
| 7 – 9 (22,909) | 2.25 (2.00-2.52) | *** | 1.92 (1.70-2.17) | *** |
| ≥ 10 (8,610) | 3.28 (2.84-3.78) | *** | 2.64 (2.2-3.08) | *** |

*S2 Table footnote -* Model 1: Adjusted for age, sex, Townsend score, ethnicity, and assessment centre location. Model 2: As model 1 and additionally adjusted for smoking status, alcohol intake frequency, BMI, and physical activity. RR = Relative risk; CI = confidence interval; n = number of participants; LTC = long-term condition; Cardiometabolic LTC = diabetes, coronary heart disease, atrial fibrillation, chronic heart failure, chronic kidney disease, hypertension, stroke/TIA or peripheral vascular disease; Respiratory LTC = asthma, chronic obstructive pulmonary disease, chronic bronchitis, emphysema, or bronchiectasis. ***p<0.01
