# Supplementary material for: Multimorbidity, polypharmacy, and COVID-19 infection within the UK Biobank cohort
Source: PLoS One. 2020 Aug 20;15(8):e0238091. doi: 10.1371/journal.pone.0238091 (PMC7440632; doi:10.1371/journal.pone.0238091)
Supplement: S3 Table — (DOCX) [file pone.0238091.s003.docx]

## S3 Table – Relative risk of COVID-19 testing by LTC category and prognostic factors.

| **Prognostic factor** | **LTC category** | **Prognostic factor subgroup** | **N** | **Relative risk**  **(95% CI)** | **P value** |
| --- | --- | --- | --- | --- | --- |
| **Sex** | 0 | Female | 80,212 | 1 (ref) |  |
|  |  | Male | 68,965 | 1.02 (0.91-1.15) | 0.72 |
|  | 1 | Female | 76,383 | 1.03 (0.92-1.15) | 0.60 |
|  |  | Male | 63,965 | 1.30 (1.07-1.34) | *** |
|  | ≥2 | Female | 77,492 | 1.38 (1.24-1.53) | *** |
|  |  | Male | 59,588 | 1.77 (1.59-1.97) | *** |
| **Age at COVID-19 test (years)** | 0 | < 65 | 16,470 | 1 (ref) |  |
|  |  | ≥ 65 | 132,707 | 0.88 (0.78-0.99) | 0.03 |
|  | 1 | < 65 | 25,029 | 1.12 (0.99-1.26) | 0.07 |
|  |  | ≥ 65 | 115,319 | 0.98 (0.88-1.10) | 0.78 |
|  | ≥2 | < 65 | 36,865 | 1.27 (1.12-1.44) | *** |
|  |  | ≥ 65 | 100,215 | 1.54 (1.40-1.70) | *** |
| **Ethnicity** | 0 | White | 138,677 | 1 (ref) |  |
|  |  | Other | 9,492 | 1.37 (1.12-1.67) | *** |
|  | 1 | White | 131,644 | 1.10 (1.01-1.20) | 0.03 |
|  |  | Other | 8,055 | 1.50 (1.22-1.83) | *** |
|  | ≥2 | White | 129,033 | 1.55 (1.42-1.68) | *** |
|  |  | Other | 7,408 | 2.04 (1.70-2.44) | *** |
| **Townsend quintile**  **(1-least deprived;**  **5-most deprived)** | 0 | 1 | 30,995 | 1 (ref) |  |
|  |  | 2 | 31,206 | 1.06 (0.87-1.29) | 0.55 |
|  |  | 3 | 30,394 | 0.98 (0.81-1.20) | 0.88 |
|  |  | 4 | 29,633 | 1.32 (1.09-1.59) | *** |
|  |  | 5 | 26,956 | 1.32 (1.09-1.59) | *** |
|  | 1 | 1 | 28,723 | 1.19 (0.98-1.44) | 0.08 |
|  |  | 2 | 28,898 | 1.24 (1.03-1.50) | 0.03 |
|  |  | 3 | 28,605 | 1.10 (0.91-1.34) | 0.34 |
|  |  | 4 | 27,950 | 1.29 (1.07-1.56) | 0.01 |
|  |  | 5 | 26,005 | 1.42 (1.18-1.71) | *** |
|  | ≥2 | 1 | 25,310 | 1.42 (1.17-1.71) | *** |
|  |  | 2 | 26,585 | 1.55 (1.29-1.86) | *** |
|  |  | 3 | 26,771 | 1.69 (1.41-2.06) | *** |
|  |  | 4 | 27,764 | 1.81 (1.52-2.16) | *** |
|  |  | 5 | 20,670 | 2.24 (1.89-2.65) | *** |
| **Smoking status** | 0 | never | 88,737 | 1 (ref) |  |
|  |  | current/previous | 59,500 | 1.18 (1.05-1.33) | *** |
|  | 1 | never | 77,915 | 1.08 (0.97-1.21) | 0.17 |
|  |  | current/previous | 61,781 | 1.32 (1.18-1.48) | *** |
|  | ≥2 | never | 68,324 | 1.47 (1.32-1.64) | *** |
|  |  | current/previous | 67,956 | 1.91 (1.72-2.12) | *** |
| **Physical activity level** | 0 | ≥ guidelines | 80,715 | 1 (ref) |  |
|  |  | < guidelines | 36,507 | 1.21 (1.05-1.40) | 0.01 |
|  | 1 | ≥ guidelines | 74,324 | 1.26 (1.09-1.46) | *** |
|  |  | < guidelines | 32,998 | 1.14 (0.96-1.36) | 0.14 |
|  | ≥2 | ≥ guidelines | 68,226 | 1.65 (1.43-2.04) | *** |
|  |  | < guidelines | 29,622 | 1.74 (1.48-1.91) | *** |
| **BMI (kg/m^2^)** | 0 | <40 | 146,986 | 1 (ref) |  |
|  |  | ≥40 | 1,094 | 1.45 (0.85-2.46) | 0.17 |
|  | 1 | <40 | 137,865 | 1.11 (1.02-1.21) | 0.01 |
|  |  | ≥40 | 1,912 | 1.48 (0.99-2.70) | 0.06 |
|  | ≥2 | <40 | 131,416 | 1.57 (1.46-1.70) | *** |
|  |  | ≥40 | 4,865 | 2.60 (2.15-3.16) | *** |
| **Systolic blood pressure**  **(mm Hg)** | 0 | <140 | 97,526 | 1 (ref) |  |
|  |  | ≥140 | 47,162 | 0.96 (0.84-1.09) | 0.72 |
|  | 1 | <140 | 78,786 | 1.12 (1.01-1.25) | 0.02 |
|  |  | ≥140 | 57,921 | 1.03 (0.92-1.16) | 0.98 |
|  | ≥2 | <140 | 68,956 | 1.51 (1.36-1.67) | *** |
|  |  | ≥140 | 63,979 | 1.55 (1.36-1.72) | *** |
| **eGFR (ml/min/1.73m^2^)** | 0 | ≥ 60 | 137,083 | 1 (ref) |  |
|  |  | < 60 | 1,270 | 1.12 (0.62-2.03) | 0.71 |
|  | 1 | ≥ 60 | 128,860 | 1.08 (1.00-1.18) | 0.06 |
|  |  | < 60 | 2,108 | 1.71 (1.19-2.45) | *** |
|  | ≥2 | ≥ 60 | 123,182 | 1.48 (1.36-1.60) | *** |
|  |  | < 60 | 4,981 | 2.98 (2.49-3.57) | *** |

*S3 Table footnote -* Models were adjusted for sex, age, ethnicity, Townsend score, smoking status, alcohol intake frequency, physical activity, BMI, and assessment centre location. LTC=long-term condition; BMI=body mass index; eGFR=estimated glomerular filtration rate; Guidelines = UK guidelines of 150 min/week moderate or 75 min/week vigorous physical activity. ***p<0.01
