# Supplementary material for: Multimorbidity, polypharmacy, and COVID-19 infection within the UK Biobank cohort
Source: PLoS One. 2020 Aug 20;15(8):e0238091. doi: 10.1371/journal.pone.0238091 (PMC7440632; doi:10.1371/journal.pone.0238091)
Supplement: Attachment — Submitted filename: Response_to_reviewers_PLOS_ONE_final.docx [file pone.0238091.s004.docx]

# Response to reviewers

Please find our point-by-point response to the reviewer’s comments below. Highlighted text below denotes new or altered text in the main manuscript.

| Reviewer comment | Authors’ response |
| --- | --- |
| Reviewer #1: This paper reports an analysis undertaken using the UK Biobank cohort to examine the relationship between multiple long-term conditions (multimorbidity) and the likelihood of testing positive to COVID-19. The aim of the paper is to contribute to the evidence base in an effort to assist in developing more robust risk stratification models to help protect those most vulnerable to COVID-19. This paper reports that multimorbidity esp when combined with socioeconomic deprivation, renal impairment and BMI of 40 or more (measured a decade or more earlier) is associated with a greater likelihood of being tested and testing positive for COVID-19. Whilst they are not unexpected findings, they make a useful contribution to the literature as we build the evidence base to support clinical decision making during the COVID-19 pandemic. | We thank the reviewer for their comment. |
| The paper could be improved by addressing these questions:  • Baseline data are reported as being collected between 2006-2010 – am I correct in thinking that the LTC status was established then? That is 10 years or more ago. If that is the case, people may have additional LTCs that you are not aware of. How have you accounted for this? The same questions should be answered for medications, physical activity, BMI, BP etc. There is one mention of this limitation in the discussion. This needs to be expanded upon and made clearer from the outset when describing the study. | We thank the reviewer for pointing out this issue. It is correct that baseline data including LTC counts were collected between 2006-2010. We agree that the LTC counts here will represent underestimates as LTCs tend to accumulate with age. We are unable to account for this other than to acknowledge this as a limitation of this dataset. However, LTC counts presented are underestimates and the associations presented are therefore also likely to be underestimates due to misclassification and regression dilution bias. This is described in the limitations section (page 20, line 390): ‘However, LTC and medication count are likely to have remained the same or increased with time and therefore our effect size estimates may be conservative.’  We describe the measurement of LTCs (page 7, line 155) and polypharmacy (page 8, line 165) as being at baseline in the methods. We have made this clearer by adding a further sentence (page 8, line 169) to state this fact again:  ‘As for LTC and polypharmacy measures, all exposures were based on assessment at the time of recruitment.’  We have added a further sentence in the strengths and limitations section to expand on this limitation (page 20, line 387):  ‘Depending on the direction and level of change since recruitment, more up-to-date data on exposures (e.g. smoking, alcohol intake, physical activity, and post code of residence) could have provided different results.’  We hope that this limitation is now clearer for readers who are interpreting our results. |
| • Can you explain how you decided the category cut-offs (e.g. age of 65yo?) | We decided to use the age cut-off used by the Centre for Disease Control: >65 years representing higher risk. (Centers for Disease Control and Prevention: Coronavirus Disease 2019 (reference number 14 <https://www.cdc.gov/coronavirus/2019-ncov/need-extra-precautions/older-adults.html>) |
| • The outcome is COVID-19 test or COVID-19 positive – is there information about whether the person was unwell or not? Can you link with death or hospitalisation data or visits to GPs? This would strengthen the paper. | We thank the reviewer for this comment and agree that a more direct measure of severity like death or hospitalisation would strengthen the paper. Unfortunately, death and hospitalisation data were not available. However, a COVID-19 test during the study period here was ipso facto a proxy for more severe disease due to the testing strategy in place at the time as only those in hospital were being tested for COVID-19. We direct the reviewer to our comment on this issue in our strengths and limitations section (page 20, line 378):  ‘Secondly, the denominator for the test group included all those who had a negative test result as well as those who were not tested at all. At the time for which COVID-19 test data are available, the strategy in the UK had been to only test those in hospital (emergency department and inpatient) settings. This means the positive COVID-19 participants are likely to have had sufficiently severe clinical signs and symptoms to justify hospital assessment and those with COVID-19 but with mild symptoms are less likely to have been tested. Our results are therefore likely to reflect the associations with more severe COVID-19 disease.’  In addition, the novelty of our work is that it is the first to show a higher risk of COVID-19 infection in those with multimorbidity. There is already another preprint looking at multimorbidity and mortality (reference number 11). |
| • Being limited to the age group of 48-86y is a significant limitation and should be discussed more fully. A key clinical question is whether and to what extent MM increases risk of COVID-19 for younger people (esp those from deprived areas), this is worth commenting on. I also found it very interesting to note that you report that if you are older than 65 and have no LTC then you are less likely (RR=0.66) to have a positive COVID-19 test – this is worthy of discussion too and what it might mean for individuals and clinicians. | We thank the reviewer for this comment. We agree it would strengthen the paper if a wider age range were available. And we agree that this is likely to be more important for more deprived populations who have higher mortality and multimorbidity at younger ages.  We have added the following to the strengths and limitations section (page 21, line 397):  ‘Finally, participants in this study were aged between 48-86 years old and associations between multimorbidity and COVID-19 may be different for younger age groups. This may be particularly important for participants from more deprived backgrounds who are more likely to have multimorbidity at a younger age.(38,39)’  We thank the reviewer for highlighting the result of those ≥65 years old having a lower risk of COVID-19. We have added the following to the results section…  Page 16, line 296: ‘Of those with no LTCs, being ≥65 years old was associated with 34% lower risk of COVID-19 compared with those <65 years old.’ |
| • Why did you exclude the 5 patients from Scotland and Wales? | We only analysed those participants who were recruited from England as test data were not available for participants currently living in Scotland and Wales. The 5 participants with test data who were recruited from Scotland and Wales must have moved or travelled to England at some point in order to have COVID-19 test data available here. We adjusted for assessment centre location in our models in an attempt to reduce confounding due to geographical location and local epidemics. In order to simplify analyses, we excluded the few who were recruited outside England but had COVID-19 data. |
| • The group who were not tested is likely to include people who did have COVID-19 but were not ill enough to qualify for testing. This should be discussed in more depth in the discussion. | Please see our response to the comment above regarding lack of outcome data that measures disease severity more directly.  We have also added the following to the strengths and limitations to expand on this issue more (page 20, line 381):  ‘It is not known if the associations identified in this study would be similar for those with milder COVID-19 disease.’ |
